# Buried remnants of the Laurentide Ice Sheet and connections to its surface elevation

**DOI:** 10.1038/s41598-018-31166-2

**Published:** 2018-09-05

**Authors:** Denis Lacelle, David A. Fisher, Stéphanie Coulombe, Daniel Fortier, Roxanne Frappier

**Affiliations:** 1 Department of Geography, Environment and Geomatics, University of Ottawa, Ottawa, ON Canada; 2 Department of Earth Sciences, University of Ottawa, Ottawa, ON Canada; 3 Department of Geography, Université de Montréal, Montréal, QC Canada

## Abstract

The Laurentide Ice Sheet (LIS) occupied a large part of North-America during the late Pleistocene. Determining the proper surface geometry and elevation of the LIS is of central importance to estimate global changes in sea-level and atmospheric circulation patterns during the late Pleistocene and Holocene. Despite largely disappearing from the landscape during the late Holocene, LIS remnants are found in the Penny and Barnes ice caps on Baffin Island (Canada) and ongoing permafrost degradation has been exposing relics of the LIS buried along its northern margin since the late Pleistocene. Here, we use the δ^18^O records of six LIS remnants and the late Pleistocene δ^18^O-elevation relation to establish ice elevation in their source area during the last glacial maximum (LGM). Contrary to some modeled reconstructions, our findings indicate an asymmetric LIS topography with higher ice on Keewatin Dome (~3200 m) and thinner ice in the prairies along the Plains divide (1700–2100 m) during LGM. The resiliency of icy permafrost to past warm intervals preserved relics of the LIS; these ice-marginal landscapes, now poised for thaw, should uncover more valuable clues about the conditions of the last major ice sheet on Earth.

## Introduction

The maximum and recessional limits of the LIS have been defined using empirical data^1^; whereas its surface geometry and elevation have been assessed from glaciological and geophysical models using a range of boundary conditions, such as past positions of its margins, ice rheology, presence of deformable beds and glacio-isostatic adjustments^2–6^. The models predict largely conflicting ice configurations, from a single dome centered near Hudson Bay^3,7^ to an ice sheet with multiple ice accumulation centers^1,4,8^, with significant differences in ice elevations (in the order of ±1000–2000 m in some places). These different LIS configurations translate into large uncertainties in modeling late Wisconsinan climate^9^ and melt-water contribution^8^. A reason for the large uncertainties in LIS topography is that the glacio-isostatic models are being calibrated to relative sea level curves and paleo lake shorelines (e.g., refs^6,10,11^) and subsequent models are adjusted as more of these data are becoming available^12^. However, to improve our assessment of the impacts of the LIS to global changes in climate and sea-level during late Pleistocene and Holocene (e.g., refs^13–15^), determination of its surface geometry and elevation from other methods is required, especially from actual remnants of the LIS.

**Figure 1:**
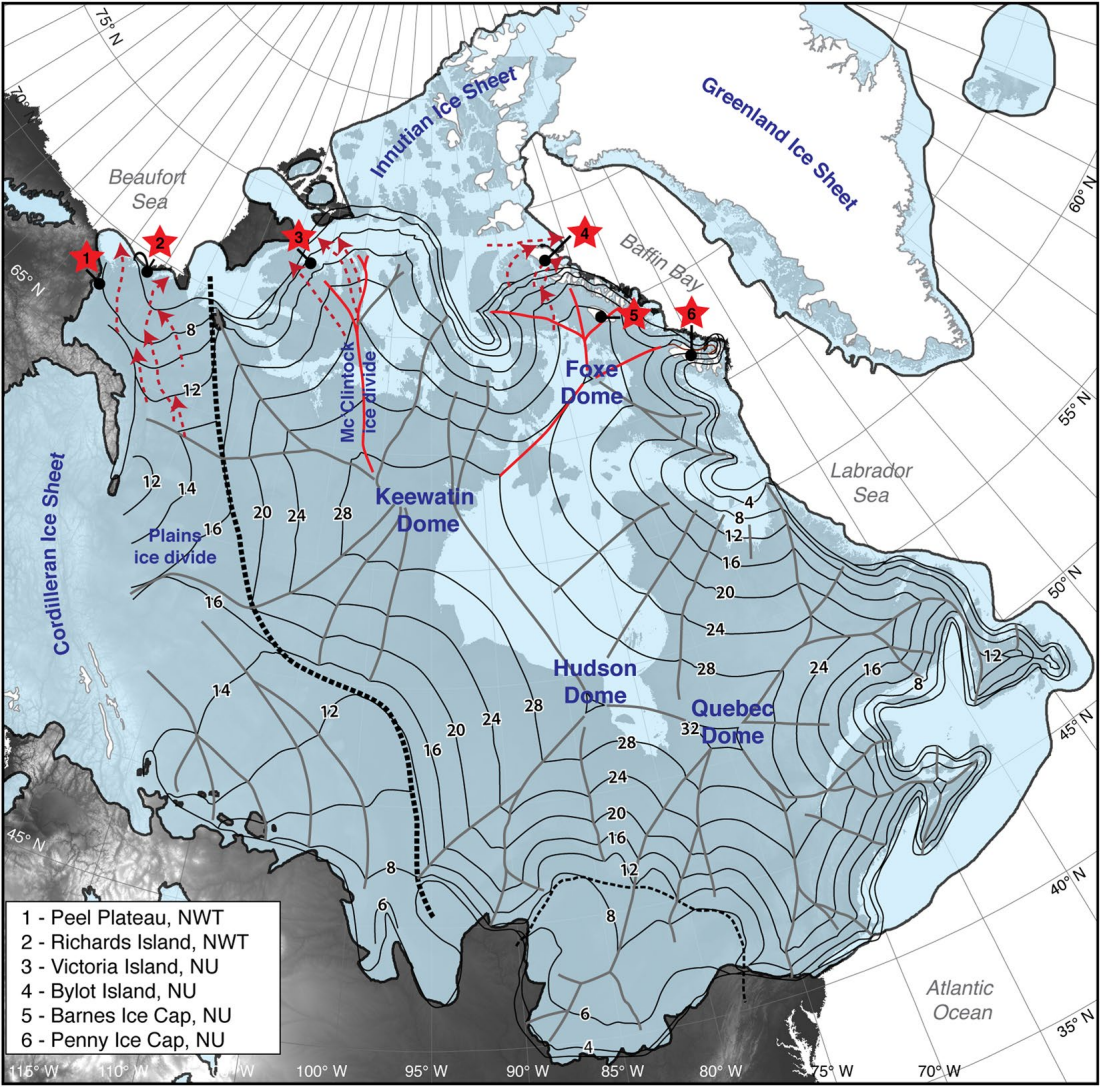
Sites of remnants of LIS ice and its reconstructed elevation during the last glacial maximum. The extent of the LIS (including ice-shelves) at last glacial maximum is derived from ref.^1^. The surface elevation of the LIS is derived from the steady-state model of ref.^2^ which is based on the empirical margins of the ice sheet (minimum-concept of ice margins and excluding ice-shelves), a simple plastic ice rheology and assumes hard-bed conditions in the Hudson Bay sector. Surface elevations are in 100′s of meters above present-day sea-level (errors are 5–7%). The thick dashed black line is the boundary between deformable beds in the Prairies and Great Lakes regions and hard beds for interior and eastern regions. The dashed red lines are inferred ice flow and source area for the four buried LIS sites. The underlying topography is from GTOPO30 digital elevation data (https://lta.cr.usgs.gov/).

In glaciated places with poor known boundary conditions (i.e., positions of ice margins), an alternative approach for the reconstruction of elevation of glaciers is to use the δ^18^O-elevation relation. This approach was used to reconstruct Holocene changes in ice surface elevation for the Greenland Ice Sheet and Agassiz Ice Cap^16,17^, with the findings used to infer an increased contribution from Greenland Ice Sheet to sea level change^17^. For the former North American late Wisconsinan ice sheets, this approach is challenged by the fact that the ice had largely disappeared from the landscape by 3–4 ka BP; nonetheless, remnants of late Wisconsinan LIS ice are still found in Penny and Barnes ice^18,19^ (Baffin Island, Canada; Fig. 1). At both Barnes and Penny ice caps, the δ^18^O_[modern-LGM]_ exceeded that in other Canadian and Greenland ice core records^20^ and could not be explained solely by changing temperatures^21^. This led refs^18,22,23^ to suggest that the ice in the north-eastern sector of the LIS originated for most of the last glacial period at higher elevation from Foxe Dome (2200–2400 m), with ice sourced during the LGM further up the flow-line and connecting it to the higher Keewatin Dome (3200 m)^23^. These elevations were derived from a glacial model using ideal ice rheology that is insensitive to unknowns parameters, the spatial extent of grounded LIS ice, yield shear stresses and the ice frozen to the bed of Hudson Bay at LGM^2^; no independent assessment of the reconstructed elevations using the δ^18^O-elevation relation was made.

In addition to the LIS remnants in Penny and Barnes ice caps, climate warming in the Canadian Arctic has been increasing thermokarst activity and buried relics of the LIS are being exposed along headwall of thaw slumps and other natural exposures^24^. Such sites have been discovered near the northern maximum limit of the LIS, including: (1) Peel Plateau, NWT, Canada; (2) Richards Island, NWT; (3) north-west Victoria Island, NU, Canada; (4) south-west Bylot Island, NU, Canada (Fig. 1). By analogy to the ice found along the margin of Greenland Ice Sheet^25,26^, the buried LIS ice near its maximum northern extent must have originated from their local accumulation centers and their δ^18^O records likely preserve details about paleo-ice elevation that can be extracted using the δ^18^O-elevation relation. Here, we use the δ^18^O records of Penny and Barnes ice caps and those of four buried LIS remnants preserved in ice-cored permafrost terrain with the late Pleistocene δ^18^O-elevation relation to estimate the LIS elevation in their source area during the LGM (see Methods for description of approach). The sites extend across the northern margin of the LIS which allows us to determine the elevation at key sectors: Foxe and Keewatin domes, Mc’Clintock and Plains ice divides. Our findings are compared to the various modeled LIS configurations during the late Wisconsinan, including Fisher-1985, Tarasov-2012, ICE5G, ICE6G, NAICE and an alternative version of the Fisher-1985 LIS elevation model^2^.

## Results

### LIS ice in Penny and Barnes ice caps (Baffin Island, NU, Canada)

**Figure 2:**
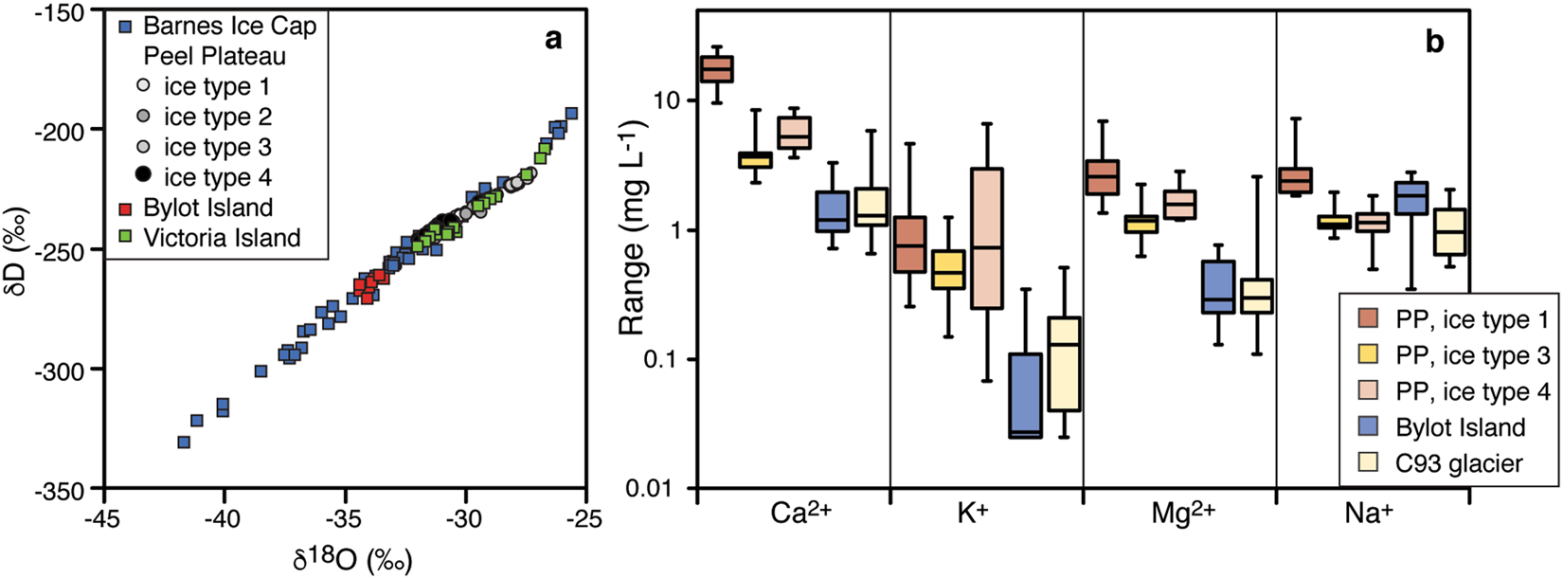
δD-δ^18^O and geochemical composition of LIS remnants. A. δD-δ^18^O scatter plot of ice from Barnes Ice Cap (from ref.^18^), the four types of buried ice at the Peel Plateau site (1: ice with mm-size spherical gas inclusions; 2: sub-vertically banded clear ice and fine sediments; 3: bubble-poor blue ice; and 4: white ice rich in spherical gas inclusions, very similar in appearance to the late Pleistocene white ice band on Barnes Ice Cap), and the buried ice on Victoria and Bylot islands. The buried ices are distributed along the Barnes Ice Cap δD-δ^18^O values, suggesting they consist of buried glacier ice. B. Range of cation concentrations (mg L^−1^) for buried ice on the Peel Plateau and Bylot Island. Also shown is cation content of C93 glacier on Bylot Island. The buried ices on the Peel Plateau and Bylot Island have cation content ~1 to 3 orders of magnitude lower than most intrasedimental ice types in permafrost^61,62^.

The δ^18^O records of Penny and Barnes ice caps have been correlated with other ice cores in Arctic Canada and Greenland and cover most of the Wisconsinan glaciation^18,19^. Near the bottom of Penny Ice Cap, late Pleistocene-age ice has average δ^18^O of −31.2 ± 1.2‰ (LGM-age ice found at 326.75 m has value of −35.9‰) with the overlying Holocene-age ice having average δ^18^O of −23.7 ± 0.6‰. On Barnes Ice Cap, late Pleistocene-age LIS ice is exposed along the western margin in a distinctive bubble-rich white band that spreads over 200 m^22^. This band of white ice has average δ^18^O of −34.3 ± 2.4‰ (with LGM-age ice having value of −41.7‰), much lower than the average of −22.6 ± 1.7‰ in the Holocene-age blue ice^18^. At Barnes and Penny ice caps, the shift in δ^18^O between modern and LGM ice (δ^18^O_[modern-LGM]_ = 12–15‰) greatly exceeds that expected due to changing air temperatures only (a shift of 3–10‰ is expected from a zonally-averaged model over latitudes of 60–80°N that includes the effect of changes in sea-ice front^21^; Fig. 2).

### Buried LIS ice in ice-cored hummocky permafrost terrain

The four buried LIS sites consist of ice that was stranded or buried shortly after the LIS reached its maximum extent at ~21–17 ka BP^1,8^, with the LIS receding from these regions by 10–14 ka BP^1^. This timing of advance and retreat of the LIS at the sites offers a relative chronology for its burial and preservation in permafrost with the receding ages providing a minimum age for the ice because of the time needed to transport ice from the accumulation center to the margin (the ice is likely LGM-age, 29–19 ka BP)^27^.

### Peel Plateau site, NWT, Canada (67°37.1′N; 135°33.4′W; 350 m a.s.l.)

On the Peel Plateau (Fig. 1), buried LIS ice was identified in ice-cored hummocky terrain near the maximum westward extent of the LIS (reached near 18 k cal yr BP)^28^ and within the Tutsieta Lake Phase recessional limit dated to 15 k cal yr BP^29^. The ice sheet had receded from the region by 13.5 k cal yr BP based on ^14^C ages obtained from remains of steppe bison near Tsigehtchic^30^. In this region, a major ice lobe flowed north-northwest along the Mackenzie Valley with the Mackenzie Through and Bear Lake ice streams likely bringing LIS ice from the more southerly Plains ice divide^2,14^.

**Figure 3:**
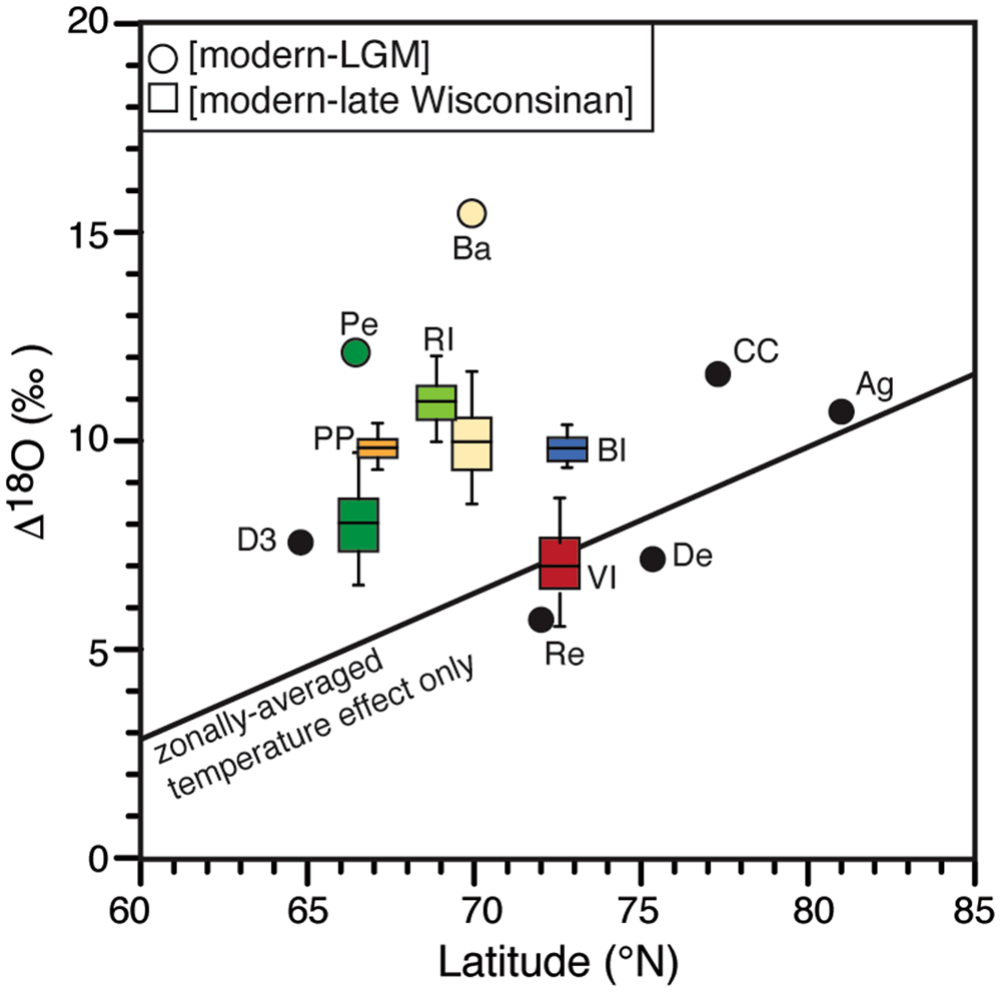
δ^18^O differences (Δ^18^O[modern–LGM] and Δ^18^O[modern-late Wisconsinan]) of LIS remnants as a function of latitude. The LIS remnants are compared to the Δ^18^O[modern-LGM] in various ice cores (from ref.^18^; Arctic Canada: Agassiz (Ag), Devon (De); Greenland: Camp Century (CC), Dye 3 (D3), Renland (Re)). The solid line is the theoretical zonally averaged Δ^18^O-latitude relation attributed solely to changes in temperatures for latitudes >55°N (from ref.^21^). The Δ^18^O of Barnes and Penny ice caps (Ba and Pe) and the four buried LIS sites (Peel Plateau: PP; Richards Island: RI; Victoria Island: VI; Bylot Island: BI) plot above the theoretical Δ^18^O-latitude relation, suggesting a non-temperature effect such as ice sourced from higher elevation during the late Wisconsinan. The Victoria Island and Bylot Island sites were differenced against modern δ^18^O in Cambridge Bay and Pond Inlet, respectively; the Peel Plateau site was differenced against modern δ^18^O in Yellowknife, which is in proximity to the region of sourced ice.

The buried ice was exposed over a 70-m wide and ca. 5 to 6 m high headwall. Leaf fragments in the soils overlying the buried ice were dated to 9534 cal yr BP. Along the headwall, four types of ice were identified: (1) clear ice with mm-size spherical gas inclusions; (2) sub-vertically banded clear ice and fine sediments; (3) bubble-poor blue ice; (4) white ice rich in spherical gas inclusions, similar to the bubble-rich white ice band on Barnes Ice Cap. The four ice types were characterized by a Ca-Na-Cl geochemical facies (Fig. 3), with concentrations being lowest in the white ice (less than 5 mg L^−1^). The white ice also has the lowest δ^18^O values (−31.2 ± 0.6‰; ranged from –32.0 to −29.5‰), whereas the other three ice types have slightly higher δ^18^O values, ranging from −31.2 to −27.3‰ (Fig. 3). The D-excess values are in the 5.0 to 9.8‰ range for the white ice (within the range of that on Penny and Barnes ice caps; Fig. S1), and slightly lower in the other ice types (in the −0.5 to 7.2‰ range). The physical, geochemical and δ^18^O and D-excess characteristics of the ice and its stratigraphic location suggest that it corresponds to late Wisconsinan age LIS ice.

### Richards Island site, NWT, Canada (69°20′; 135°18′W; 0 m a.s.l.)

On Richards Island in the Mackenzie Delta region (Fig. 1), the LIS advanced during the Toker Point Stade as part of a major Mackenzie Valley ice lobe before fanning out across the Yukon Coastal Plains, Richards Island and the Tuktoyaktuk Coastlands^31^. The LIS likely receded from the area by 14–13 ka BP^32^.

Along the shore of YaYa Lake, massive ice was identified by radar and borehole drilling beneath 7-m of glaciofluvial sand^33^. The ice had δ^18^O values in the −34.1 to −30.6‰ range (−32.4 ± 1.1‰) with low cation contents. Together with the stratigraphic context, the ice was inferred by ref^33^ to be buried LIS ice (although a segregated ice origin could not be fully ruled out by the authors).

### Victoria Island site. (73°11′N; 113°59′W; 40 m a.s.l.)

On the Prince Albert Peninsula on north-west Victoria Island, late Wisconsinan tills are cored by buried LIS ice^34^. Former ice margins suggest that the north-west sector of Victoria Island represented the maximum extent of the LIS^8^; however, a revision of the extension of the ice based on ^14^C of marine molluscs suggest that the LIS extended across Banks Island^35^, such that northern Victoria Island and the Jesse Moraines along the east coast of Banks Island represent a recessional phase of the LIS^36^. The LIS likely occupied north-west Victoria Island near 21–18 ka BP and the ice had receded by 14–12 ka BP^1,36^. On the Prince Albert Peninsula, the LIS originated from the Mc’Clintock ice divide and was likely transported by the Mc’Clintock Channel and possibly the Collinson Inlet ice streams^14^.

Near Loch Point on Prince Albert Peninsula (Fig. 1), ref.^34^ identified buried LIS ice beneath 1-m thick till extending 60–450 m laterally. The englacial ice, which contained a small amount of dispersed sediments, had a sub-vertical banding with alternating bubble-rich and bubble-free ice and its δ^18^O averaged −30.1 ± 1.4‰ (ranged from −32 to −27.5‰; Fig. 3).

### Bylot Island site. (73°09′N; 79°57′W; 25 m a.s.l.)

Buried LIS ice was discovered in the Qarlikturvik Valley on southwest Bylot Island^37^. The late Wisconsinan LIS limit was first positioned south of Bylot Island and it was suggested that alpine glaciers, similar in size to those present today, occupied Bylot Island during that time^38^. However, recent mapping by ref.^39^ provided a re-interpretation of LIS extent and it is now suggested that late Wisconsinan LIS ice filled Navy Board Inlet (as evidenced from mega-scale glacial lineations on the sea-bed) with ice flowing northward from the Lancaster Sound ice stream^40^. Radiocarbon age of shells in nearby marine sediments indicate the region was deglaciated near 9860 ± 140 ^14^C BP^41^.

The exposed buried ice in the valley was discovered beneath an ice-contact stratified drift^37^. The ice had a clear to whitish appearance, similar to the late Pleistocene-age white ice band on Barnes Ice Cap. The buried ice contained many small spherical and coalescing gas inclusions. The ice was dominated by Ca^2+^, Na^+^, Mg^2+^ cations and their concentration was low and within the range of ice sampled from the nearby C-93 glacier (Fig. 3). The δ^18^O of the buried ice ranged from −34.4 to −33.4‰ (average of −34.0 ± 0.4‰; Fig. 2) and D-excess averaged 6.6 ± 2.5‰, both similar to values obtained from Barnes Ice Cap (Fig. S1).

## Discussion

The late Wisconsinan δ^18^O records of Penny and Barnes ice caps and the four buried LIS ice range from −34.3 ± 2.4‰ to −30.1 ± 1.4‰. These values are 7–14‰ lower than amount weighted modern values in precipitation at nearby sites (Yellowknife, NWT: −20.9‰; Inuvik, NWT: −24.1‰; Cambridge Bay, NU: −23.3 ± 0.7‰; Pond Inlet, NU: −23.8‰; ref.^42^). Further, the ~4‰ difference between the δ^18^O record of the LIS remnants cannot solely be explained by regional temperatures or major moisture source differences because the δ^18^O composition of modern precipitation is strongly zonal and varies by less than 1‰ along latitudes of 67–72°; late Pleistocene δ^18^O precipitation records from 154 empirical proxy data showed a similar zonal distribution^43^. However, like Penny and Barnes ice caps^20^, the difference in Δ^18^O_[modern-late Wisconsinan]_ for the buried LIS sites is higher than expected for their latitudes (Fig. 2). Therefore, a more likely explanation of the differing δ^18^O records between the LIS remnants, including their higher than expected δ^18^O shift between late Wisconsinan and modern conditions, is differences in ice elevation in their source areas. On ice sheets and ice caps, precipitation accumulates in the catchment area above the equilibrium line and is transferred to lower elevation along flow lines; this occurs while maintaining the δ^18^O stratigraphy. This was demonstrated on the Greenland Ice Sheet where a horizontal δ^18^O transect at the edge of the ice sheet was nearly identical (with a ~1–2‰ variation) to a deep ice core at the head of its flow line near the center of the ice sheet (Pakitsoq site and GISP2 ice core)^25,26^; such ice transport and preservation of δ^18^O record was also incorporated in 3D isotope stratigraphy models^44^ (Fig. S1). Therefore, the δ^18^O records of the buried LIS ice near its maximum northern extent likely preserve details about ice surface elevation in their source areas and this information can be extracted from the δ^18^O-elevation relation.

**Figure 4:**
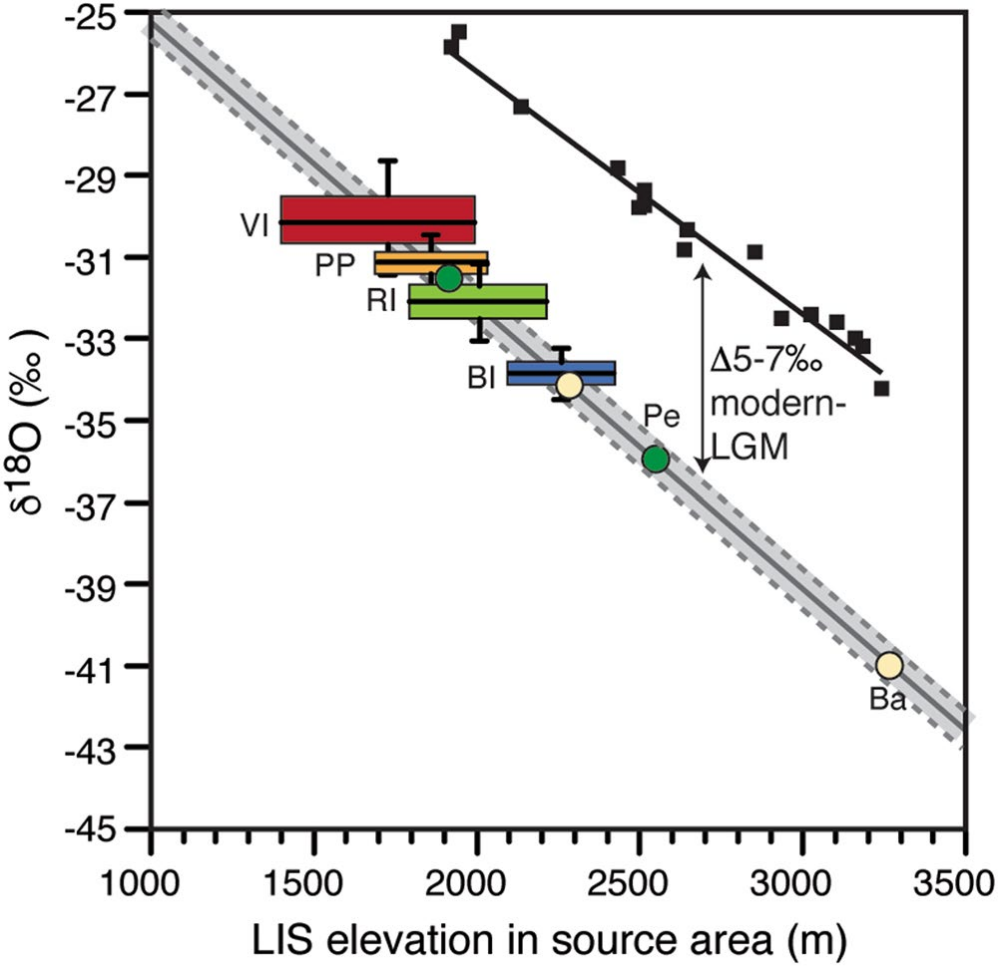
δ^18^O composition of LIS remnants and connections to its elevation. δ^18^O composition of remnants of LIS and inferred elevation in their source area during the LGM. Solid black line represents the modern δ^18^O-elevation relation established from a series of shallow ice cores on Greenland (−0.62 ± 0.03‰ per 100 m; refs^47,48^); Grey line and bar is the late Pleistocene δ^18^O-elevation relation (−0.67 ± 0.03‰ per 100 m) and corrected for the 5–7‰ offset due to cooler temperature only. Ba: Barnes Ice Cap; Pe: Penny Ice Cap; BI: Bylot Island site; VI: Victoria Island site; RI: Richards Island site; PP: Peel Plateau site.

The δ^18^O-elevation relation is largely temperature dependent^45^. Globally, a modern slope of about −0.28 to −0.33‰ per 100 m is observed^45,46^, but a series of shallow ice cores from Greenland Ice Sheet define the modern δ^18^O-elevation relation at −0.62 ± 0.03‰ per 100 m^47,48^. Similar δ^18^O-elevation slopes were measured along the east coast of Queen Elizabeth Islands (high Arctic Canada; −0.65 to −0.64‰ per 100 m; ref.^49^). The modern δ^18^O-elevation slope cannot be directly transferred to different climate periods, but empirical proxy data suggest that the δ^18^O-elevation relation during the late Pleistocene changed by −0.05‰^43^, providing a late Pleistocene δ^18^O-elevation relation of −0.67 ± 0.03‰ per 100 m. The increase in slope value during the colder late Pleistocene is similar to that reported from modern-day Antarctica (−0.68‰ per 100 m; ref.^50^). Applying the late Pleistocene δ^18^O-elevation correction and accounting for the 5–7‰ shift in δ^18^O of precipitation during the late Wisconsinan along the latitudes of our sites (a latitudinal-dependent offset that accounts for the effect of cooler temperature and shift in sea-ice front during the last glacial period^21^) allows us to define the LIS surface elevation in their source area during the late Wisconsinan (Fig. 4). For Penny Ice Cap, the late Wisconsinan and LGM δ^18^O values (−31.2 ± 1.2‰ and −35.9‰, respectively) suggest surface ice elevations of 1900–2000 m and 2500 ± 100 m. For Barnes Ice Cap, the late Wisconsinan and LGM δ^18^O values are much lower (−34.3 ± 2.4‰ and −41.7‰, respectively), suggesting surface ice elevations for most of the last glacial period of 2200–2400 m and near 3200 ± 100 m during the LGM (Fig. 1). For the buried LIS preserved along southwest Bylot Island (δ^18^O = −34.0 ± 0.4‰), the ice likely originated at similar elevation to most of the last glacial ice preserved in nearby Barnes Ice Cap (2150–2350 m). The ice at Barnes and Penny ice caps and on south-west Bylot Island would have been sourced from Foxe Dome for much of the late Wisconsinan, hundreds of km from their modern location. The very low values near −40‰ on Barnes Ice Cap and near −36‰ on Penny Ice Cap would suggest ice sourced at LGM at higher elevation and connecting it to the 3200 ± 100 m Keewatin Dome^23^ (Fig. 1). Similar low δ^18^O values (−38‰) were measured from foraminifer shells in the Gulf of Mexico and linked to LIS meltwater sourced from the Keewatin Dome draining through the Mississippi River watershed^51^. On north-west Victoria Island, the buried LIS ice (δ^18^O = −30.1 ± 1.4‰) that flowed from the Mc’Clintock ice divide likely originated from ice sourced at surface elevations of 1500–1900 m. Finally, the buried LIS ice on the Peel Plateau (δ^18^O = −31.2 ± 0.6‰) and Richards Island (δ^18^O = −32.4 ± 1.1‰) suggests ice sourced from the Plains ice divide at surface elevations of 1700–2100 m. The Peel Plateau and Mackenzie Delta region have its highest divide at the Plains divide because the transition between the hard-frozen beds of the Canadian Shield and the deforming soft-beds of the prairies translates into a very sharp change in flowline direction (the dashed line in Fig. 1).

**Figure 5:**
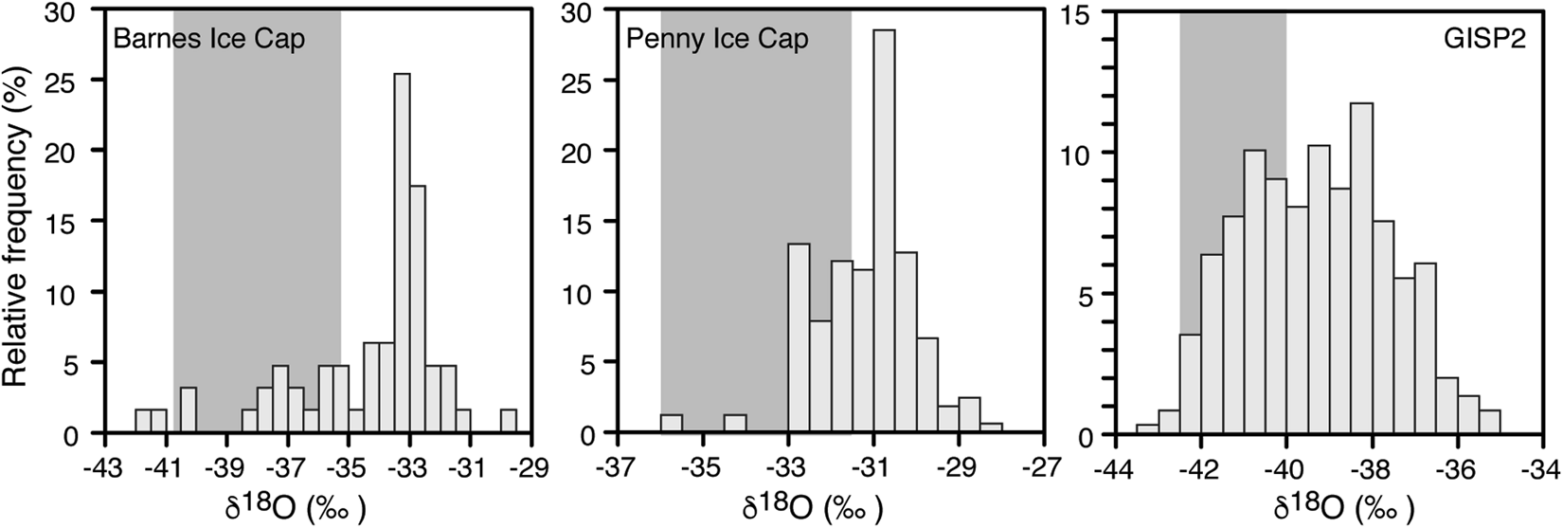
Histograms of Penny and Barnes ice caps and GISP2 δ^18^O records during last glacial period. Vertical grey bar shows range of δ^18^O values during LGM (29–19 ka BP; ref.^27^) and the sum of relative frequency of δ^18^O values for Penny and Barnes ice caps and GISP2 is 36, 28, 36%, respectively. Values are in 0.5‰ bins. Data from refs^18,23,63^.

The δ^18^O records from Penny and Barnes ice caps have a sufficient chronological control, such that we can infer ice surface elevations at these sites for the late Wisconsinan and LGM with good confidence. The buried LIS ice in the ice-cored permafrost terrains near the maximum extent of the LIS lack a robust chronological control due to challenges of obtaining reliable ages from ice itself; however, the timing of advance and retreat of the LIS at these localities indicates that the ice is of late Wisconsinan-age and most likely LGM-age. To test the effect of random sampling of the buried LIS ice and if it may represent LGM conditions (29–19 ka BP)^27^, we performed a frequency distribution analysis of the late Pleistocene age δ^18^O records of GISP2, Penny and Barnes ice caps (Fig. 5); the likelihood of randomly sampling ice with δ^18^O values in the range of LGM-age ice is high (28–36%). Additionally, the variance in δ^18^O for the late Wisconsinan ice was assessed for the GISP2 and Penny δ^18^O records and standard deviations of 1.1 to 1.4‰ were calculated. As such, random sampling of buried late Wisconsinan ice does not have δ^18^O values that would deviate greatly from LGM-age ice (these would translate into errors of <200 m in our paleo-elevations reconstruction). Increasing the uncertainties in δ^18^O to 3‰ would translate into an elevation error of 500 m.

Together, the paleo-elevation reconstruction derived from the δ^18^O records of six LIS remnants sites indicate an asymmetric LIS during the LGM, with ice surface elevations near 2200–2400 m on Foxe Dome, 3200 ± 100 m on the Keewatin Dome and 1700–2100 m along the Plains ice divide (Fig. 1). Overall, our reconstruction of ice surface elevation from northern sectors of the LIS near LGM is consistent with the Fisher-1985 model, using both minimum and maximum grounded ice margin extents (Figs 1 and S2), and the Tarasov-2012 model^5^. These models predicted within errors similar surface elevations for the Foxe and Keewatin domes, and the Mc’Clintok and Plains ice divides. Our δ^18^O-elevation reconstruction of shallow ice along the Plains ice divide is also consistent with: (1) with the mapped glacial limits in the south-eastern Mackenzie Mountains where the LIS reached maximum elevation of ~1550 m^52^ (descending in elevation to 750 m in the Richardson Mountains)^28^; (2) surface exposure age near 21 kyr for elevations >1400 m in the western Mackenzie Mountains^53^; and (3) reported δ^18^O values from other buried glacier ices and segregated ices in northwestern Canada, which are all greater than −34‰ (mainly in the −32 to −28‰ range)^54^. The ICE5G, ICE6G and NAICE models generally predicted too much ice in the Keewatin Dome and/or Plains ice divide. Given some uncertainties in our approach and in geophysical models, we highlight differences in the order of 1000 m between these various paleo-elevation reconstructions at LGM. For example, the ICE5G model^55^ has similar ice elevations for the Foxe Dome and along the Mc’Clintock and Plains ice divides, but it has a much thicker Keewatin Dome (~4000 m). Conversely, the ICE6G model^6^ has similar ice elevations for the Keewatin and Foxe domes, but increased ice elevation along the Plains ice divide (2800–3200 m). The NAICE model^4^ also has much higher ice elevation along the Plains ice divide (~2500–3000 m).

The concept of using δ^18^O records from LIS remnants in glaciers and permafrost to derive surface elevation of the LIS provides valuable constraints to modeling efforts. Our δ^18^O-derived paleo-elevations have decent fit with the Fisher-1985 glacial model and the Tarasov-2012 glacio-geophysical model and supports the estimated LIS ice volume at LGM of ~21.1 × 10^6^ km^3^ (ref.^2^; increases to ~25.9 × 10^6^ km^3^ if the maximum-concept of ice margins at LGM is used). Some of the latest modeled glacio-geophysical LIS elevation reconstruction are generally found to be over-estimating its surface elevation near the LGM on the Keewatin Dome and along the Plains ice divide, although these may also relate to uncertainties in our ice-age estimates and the dynamic nature of the LIS. There are probably other remnants near former ice-marginal extents of past ice sheets in North-America and Siberia that contain information that would help reconstruct their interior geometry and global-scale impacts. Given that the ongoing climate warming and associated thermokarst is exposing new sites that potentially contain LIS relics in permafrost^24^, these should be found and sampled before they melt away as they contain key information about the conditions prevailing for the last major ice sheet on Earth (i.e., trapped gases, impurities).

## Methods

### Identifying buried LIS ice

Distinguishing between buried glacial ice and other massive ice types in permafrost is usually based on cryostratigraphy and combines detailed physical, geochemical and isotopic measurements of the ice and may also include analyses of occluded gases, while its chronology usually relies on the age of the surrounding sediments^28^.

#### Sampling

The buried ice on the Peel Plateau and Bylot Island were sampled in summer 2016 and 2012, respectively. At both sites, a cryostratigraphic approach was used to describe the exposed ice and the overlying sediments. Samples of ice were collected using either an ice pick or a portable core-drill. All samples were shipped and stored in our laboratories until analyses.

#### Water isotope analysis

The stable isotope ratios of oxygen (^18^O/^16^O) and hydrogen (D/H) were determined using a Los Gatos Research high-precision liquid water analyzer coupled to a CTC LC-PAL autosampler. The results are presented using the δ-notation (δ^18^O and δD), where δ represents the parts per thousand differences for ^18^O/^16^O or D/H in a sample with respect to Vienna Standard Mean Ocean Water (VSMOW). Analytical reproducibility for δ^18^O and δD was ±0.3‰ and ±1‰, respectively.

#### Geochemical analysis

The concentration of major cations in the ices was determined by inductively coupled plasma optical emission spectrometry (Vista Pro ICP-OES) at the University of Ottawa. Solutes are expressed in mg L^−1^ and analytical reproducibility was ±1%.

### Inferring LIS surface elevation from the δ^18^O records of its remnants

The approach used to derive the surface elevation of the LIS during the late Wisonsinan for sites along its northern margin combines: (1) the stable isotope-based paleo-altimetry method used to reconstruct Holocene changes in surface elevation of the Greenland Ice Sheet and Agassiz Ice Cap^16,17^; (2) the δ^18^O-elevation relation adjusted for late Pleistocene conditions^43^; and (3) the concept of an ice sheet flow-line model that predicts that the margins of ice sheets contain ice that originate from their local accumulation area at much higher elevation^25,44,56^.

#### Ice flow in ice sheets

Ice sheet flow models that predicts that the margins of glaciers contain ice that originate from their local accumulation area at much higher elevation^25,44,56^. The latter was observed on Greenland Ice Sheet where the δ^18^O record of ice sampled along a horizontal transect from the western edge (Pakitsoq site) was remarkably similar to that of the GISP2 δ^18^O ice core record situated near the center of its accumulation area due to ice resurfacing in the ablation zone^25,26^. The ice-flow on Greenland Ice Sheet was successfully modeled using a 3D transport model fitted with δ^18^O data from ice cores^44^. In fact, both Pakitsoq and Barnes Ice Cap exposed late Pleistocene-age ice near their margins over a horizontal distances of ~200–500 m; with Holocene-age ice above it (Fig. S1).

#### Holocene to late Pleistocene change in δ^18^O-elevation relation

The δ^18^O-elevation relation is largely temperature dependent^45^; globally a modern slope of about −0.28 to −0.33‰ per 100 m is reported, but it increases in Arctic regions with reported values of −0.62 ± 0.03‰ per 100 m^47,48^ on Greenland Ice Sheet and of −0.65 to −0.64^49^ on the east coast of the Queen Elizabeth Islands (high Arctic Canada). The steeper slope in Arctic region is attributed to colder-drier conditions and near adiabatic lapse rate with little mixing of air masses as they gain elevation; this is particularly the case on large ice sheets. In the lower latitudes, vertical advective mixing is much stronger and the mixing of air masses reduces the δ^18^O-elevation slope.

The δ^18^O of precipitation during the late Wisconsinan is largely affected by changes in air temperature, site elevation, seasonal distribution in amount of precipitation and atmospheric circulation, the latter two relate to changes in moisture source. The effect of a change in zonally-averaged air temperature during the late Wisconsinan, including a shift in the position of sea-ice front, was associated with a latitudinal-dependent shift of 3–10‰ in δ^18^O of precipitation, with the higher latitudes experiencing a greater shift^21^. This trend was also observed in 154 late Pleistocene δ^18^O proxy records^43^. The modern δ^18^O-elevation slope cannot be directly transferred to other and different climate periods, such as the cooler late Pleistocene. However, based on empirical data, ref.^43^ suggested that the global δ^18^O-elevation relation during the late Pleistocene changed by −0.05‰ per 100 m. We therefore use a late Pleistocene δ^18^O-elevation relation of −0.67 ± 0.03‰ per 100 m for Arctic regions. This late Pleistocene δ^18^O-elevation slope is nearly identical to that observed in the colder Antarctica (−0.68‰ per 100 m; ref.^50^). Changes in seasonal distribution in amount precipitation and atmospheric circulation can be assessed from the D-excess parameter, which can provide clues into changes in distance to moisture source (increasing values with increasing distance from moisture)^57^. Examining the D-excess record of Penny Ice Cap (Fig. S1) shows values during the late Wisconsinan that are similar in range to that during the Holocene, suggesting little change in moisture source. That is expected because precipitation along Baffin and Bylot islands (and the east coast of the Queen Elizabeth Islands) largely originates from nearby Baffin Bay with the air masses experiencing adiabatic cooling during uplift over the mountain ranges followed by isobaric cooling in the interior (i.e., rainout at constant condensation altitude with Rayleigh-type distillation)^17,49^. However, potential changes in moisture source during the late Wisconsinan in the north-central (i.e., Victoria Island site) and north-western (i.e., Peel Plateau and Richards Island sites) sectors of the LIS have more uncertainty. The D-excess values of the buried white ice on the Peel Plateau are within the range of that on Penny and Barnes ice caps and also of that calculated in modern-day precipitation at nearby sites (Inuvik: 14.9 ± 7.6‰; Cambridge Bay: 9.6 ± 4.8‰)^58^. Therefore, if there was a change in distance to moisture source, it is not reflected in the D-excess values. However, to truly assess source distribution changes, one would have to do a trajectory tracing of ancient climates over the proposed ice sheet geometries. The biggest unknown relates to the age of the sampled buried ices. We ascribed to the four buried LIS ice a near LGM-age (29–19 ka BP)^27^ based on the existing chronologies of the timing of advance and retreat of the LIS in these areas. The LIS reached its maximum extent at ~21k-17 ka BP^1,8^ and had receded from these regions by 10–14 ka BP^1^. This timing of retreat of the LIS at the sites provides a minimum age constraints for the sampled ice with the actual age at each site being dependent on the ice flow rate in the ice sheet. The uncertainty in the age of the sampled buried LIS sites is the biggest uncertainty when comparing reconstructed paleo-elevations derived from the δ^18^O-elevation relation to those from various glaciological and geophysical models that have reconstruction for specific times because the LIS topography is evolving over time.

### Modeling LIS surface elevation with minimum- and maximum-concept ice margins

The reconstruction of late Wisconsinan LIS surface elevation at 18 ka is from ref.^2^. It uses a simple plastic ice model that is insensitive to unknown parameters and uses as inputs the margins of the ice sheet, present-day topography and an assumed yield shear stress. References^59,60^ showed that elevation estimates using this model are good to 6% on present-day Greenland, with ridges and domes having smaller errors. No assumptions are made in advance about ice divides and accumulation centers. At the time of its publication in 1985, some uncertainty still existed about northern and northeastern LIS margins, thus ref.^2^ presented the LIS surface elevation for the minimum-concept of grounded ice margins. The maximum-concept of ice margins was produced at that time also and is shown in Fig. S2. It uses all the same inputs as the minimum-concept model. Increasing the ice margins allow for slightly higher LIS elevations and for a higher ice volume (25.9 × 10^6^ km^3^). Note that the Cordilleran and Innuitian ice sheets have not been included in these reconstructions so their volumes are not included.

## Electronic supplementary material

Supplementary information
