# Supplementary material for: Buried remnants of the Laurentide Ice Sheet and connections to its surface elevation
Source: Sci Rep. 2018 Sep 5;8:13286. doi: 10.1038/s41598-018-31166-2 (PMC6125386; doi:10.1038/s41598-018-31166-2)
Supplement: Supplementary file 1 — Supplementary information [file 41598_2018_31166_MOESM1_ESM.pdf]

Supplementary Material

Buried remnants of the Laurentide Ice Sheet and connections to its surface elevation

Denis Lacelle[1], David A. Fisher[2], Stéphanie Coulombe[3], Daniel Fortier[3], Roxanne Frappier[1]

[1]Department of Geography, Environment and Geomatics, University of Ottawa, ON, Canada
[2]Department of Earth Sciences, University of Ottawa, ON, Canada
[3]Department of Geography, Université de Montréal, QC, Canada

Content

1. Data availability
2. Supplementary table 1. Peel Plateau site: δD, $\delta^{18}O$ and D-excess data.
3. Supplementary figure 1. Schematic diagram of ice flow in ice sheet and associated $\delta^{18}O$ records near center of accumulation and margins.
4. Supplementary figure 2. Sites of remnants of LIS ice and its reconstructed elevation during the last glacial maximum (using maximum-concept ice margins).
5. References.

Data availability

- *Penny Ice Cap.* The $\delta^{18}O$ record of Penny Ice Cap is available at: https://www.ncdc.noaa.gov/paleo/study/2474.
- *Barnes Ice Cap.* The $\delta^{18}O$ and D-excess records of Barnes Ice Cap are available in ref.[1]
- *Victoria Island site*. The $\delta^{18}O$ record is available from ref.[2]
- *Bylot Island site.* The $\delta^{18}O$ and D-excess records are available at Nordicana D (http://www.cen.ulaval.ca/nordicanad/index.aspx); Nordicana D40 (DOI: 10.5885/45564CE-8A9A55185FBD4283).
- Richards Island site. The $\delta^{18}O$ record is available from ref.[3]
- *Peel Plateau site.* The $\delta^{18}O$, δD and D-excess records are available at Table S1.

Table S1. Peel Plateau site: δD, $\delta^{18}O$ and D-excess data.

Ice type	$\delta^{18}O$ (‰)	δD (‰)	D-excess (‰)	Ice type	$\delta^{18}O$ (‰)	δD (‰)	D-excess (‰)
1	-29.7	-233.2	4.4	3	-28.1	-224.0	1.1
1	-29.5	-231.3	4.8	3	-28.2	-223.6	2.0
1	-29.0	-228.6	3.5	3	-28.0	-222.7	1.4
1	-29.5	-230.3	5.4	3	-27.9	-222.5	0.5
1	-30.0	-234.7	5.4	3	-27.9	-223.4	0.2
1	-30.2	-235.8	5.5	3	-28.2	-223.9	1.6
1	-30.3	-235.8	6.5	3	-27.3	-218.5	0.0
1	-31.1	-241.3	7.1	3	-28.2	-223.2	2.0
1	-30.9	-240.7	6.7	3	-27.6	-220.5	0.6
1	-30.7	-239.2	6.2	3	-27.8	-222.8	-0.3
1	-30.3	-236.0	6.3	3	-27.9	-222.6	0.3
1	-30.4	-236.4	6.9	3	-27.5	-219.9	0.0
1	-30.4	-238.3	4.9	3	-27.5	-220.3	-0.6
1	-30.8	-240.2	5.9	3	-27.9	-222.1	0.8
1	-30.7	-238.7	7.2	4	-31.1	-240.0	9.1
1	-31.2	-242.5	6.9	4	-32.0	-246.1	9.8
1	-30.2	-236.1	5.8	4	-31.0	-239.7	8.4
1	-29.7	-232.0	5.3	4	-29.5	-230.8	5.0
2	-29.7	-232.5	5.1	4	-31.0	-238.6	9.0
2	-29.1	-229.0	3.8	4	-31.3	-241.0	9.2
2	-30.0	-235.4	4.6	4	-31.5	-243.1	8.9
2	-31.3	-245.6	4.8	4	-31.3	-241.5	8.9
2	-30.9	-243.0	4.2	4	-31.9	-247.1	8.1
2	-30.6	-240.5	4.3	4	-31.7	-244.2	9.3
2	-30.5	-238.6	5.4	4	-31.1	-242.4	6.6
2	-29.4	-234.7	0.5	4	-30.7	-238.4	6.8
2	-28.7	-227.2	2.4				

Ice type	Description
1	clear ice with mm-size spherical gas inclusions
2	sub-vertically banded clear ice and fine sediments
3	bubble-poor blue ice
4	white ice rich in spherical gas inclusions

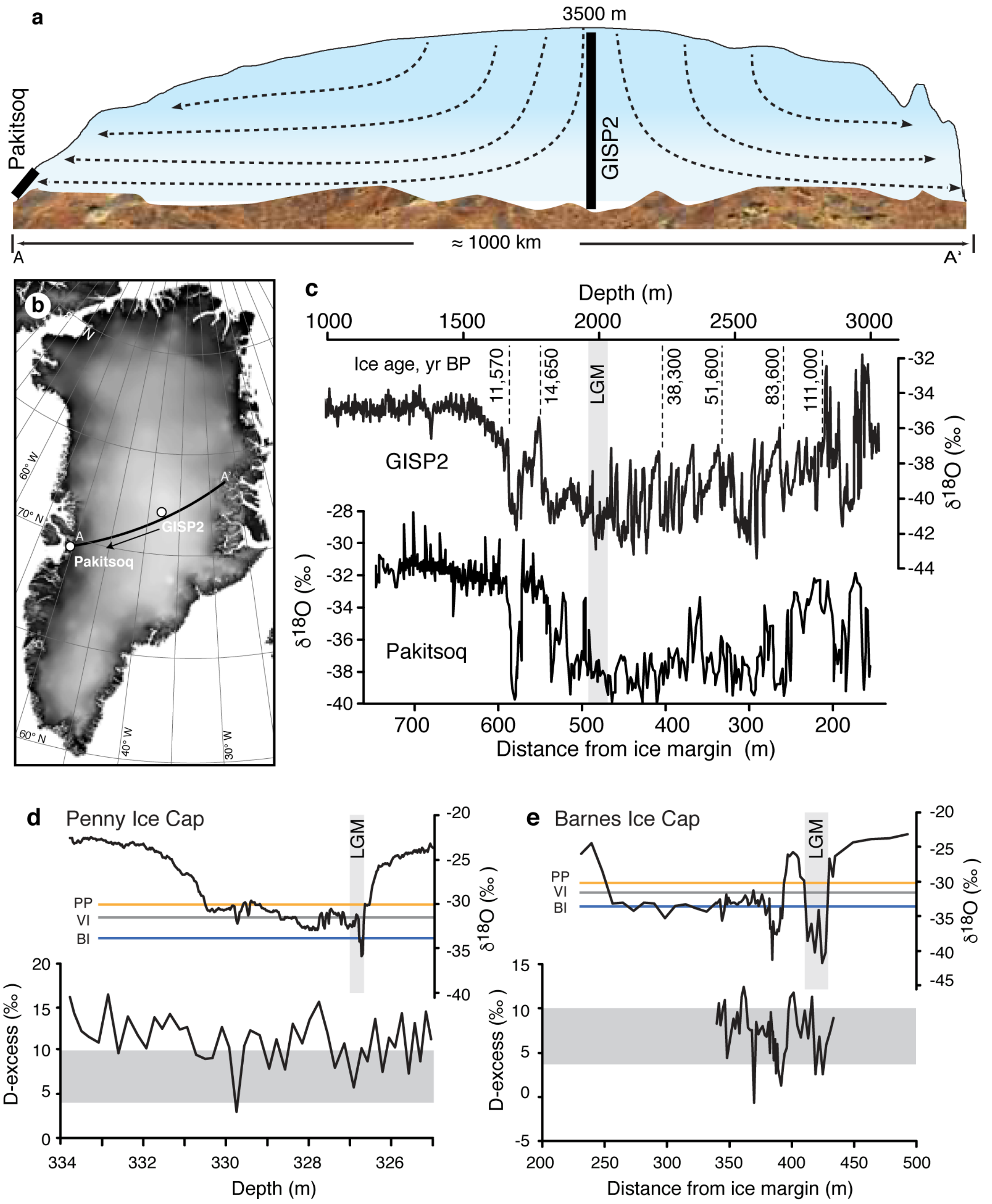

Figure S1 | Schematic diagram of ice flow in ice sheet and associated $\delta^{18}O$ records near center of accumulation and margins. A. Topography of Greenland Ice Sheet along cross-section shown in (B) and location of GISP2 and Pakitsoq ice records. Topography is derived from GTOPO30 digital elevation data (https://lta.cr.usgs.gov/). C. GISP2 and Pakitsoq $\delta^{18}O$ records for the last glacial period (from ref.[4,5]). Both records are remarkably similar, suggesting that ice along the margin of ice sheet contain ice that originate from their source area. D-E) Penny and Barnes ice caps $\delta^{18}O$ and D-excess records for the last glacial period (from refs.[1,6]). Shown for comparison are the average $\delta^{18}O$ and range of D-excess for the three buried LIS ice (horizontal grey bars); PP: Peel Plateau; VI: Victoria Island (from ref.[2]); BI: Bylot Island.

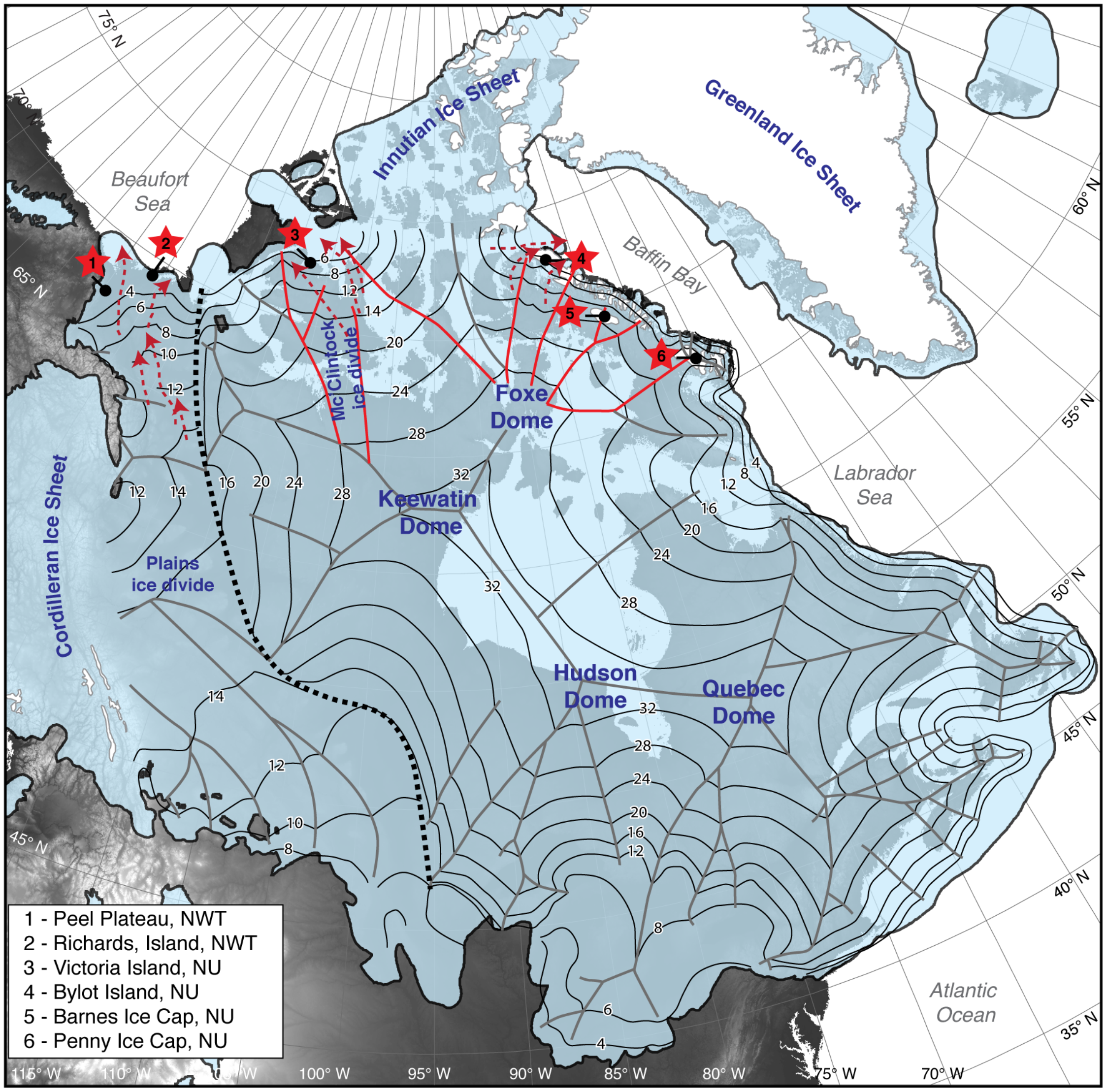

Figure S2 | Sites of remnants of LIS ice and its reconstructed elevation during the last glacial maximum (using maximum-concept ice margins). The extent of the LIS (including ice-shelves) at last glacial maximum is derived from ref.[7]. The surface elevation of the LIS is derived from the steady-state model of ref.[8] which is based on the empirical margins of the ice sheet (maximum-concept of ice margins and excluding ice-shelves), a simple plastic ice rheology and assumes hard-bed conditions in the Hudson Bay sector. Surface elevations are in 100's of meters above present-day seal-level (errors are 5-7%). The dashed black line is the boundary between deformable beds in the Prairies and Great Lakes regions and hard beds for interior and eastern regions. The dashed red lines are inferred ice flow and source area for the three buried LIS sites. The underlying topography is from GTOPO30 digital elevation data (https://lta.cr.usgs.gov/).

References

1. Zdanowicz, C. M., Fisher, D. A., Clark, I. D. & Lacelle, D. An ice-marginal δ18O record from Barnes Ice Cap, Baffin Island, Canada. *Ann. Glaciol.* **35,** 145–149 (2002).
2. Lorrain, R. D. & Demeur, P. Isotopic evidence for relic Pleistocene glacier ice on Victoria Island, Canadian Arctic Archipelago. *Arct. Alp. Res.* **17,** 89–98 (1985).
3. Dallimore, S. R. & Wolfe. Massive ground ice associated with glaciofluvial sediments, Richards Island, N.W.T., Canada. in *5th International Conference on Permafrost* 132–137 (Tapir Publisher, 1988).
4. Reeh, N., Oerter, H. & Thomsen, H. H. Comparison between Greenland ice-margin and ice-core oxygen-18 records. *Ann. Glaciol.* **35,** 136–144 (2002).
5. Grootes, P. M. & Stuiver, M. Oxygen 18/16 variability in Greenland snow and ice with 10−3- to 10 5-year time resolution. *J. Geophys. Res.* **102,** 470,26426-26455 (1997).
6. Fisher, D. A. *et al.* Penny Ice Cap cores, Baffin Island, Canada, and the Wisconsinan Foxe Dome connection: Two states of Hudson Bay ice cover. *Science (80-.).* **279,** 692–696 (1998).
7. Dyke, A. S. & Prest, V. K. Late Wisconsinan and Holocene History of the Laurentide Ice Sheet. *Géographie Phys. Quat.* **41,** 237 (1987).
8. Fisher, D. A., Reeh, N. & Langley, K. Objective Reconstructions of the Late Wisconsinan Laurentide Ice Sheet and the Significance of Deformable Beds. *Géographie Phys. Quat.* **39,** 229 (1985).
